# A DNA microarray for the authentication of giant tiger prawn (*Penaeus monodon*) and whiteleg shrimp (*Penaeus* (*Litopenaeus*) *vannamei*): a proof-of-principle

**DOI:** 10.1007/s00216-021-03440-2

**Published:** 2021-06-11

**Authors:** Kristina Kappel, Joanna Fafińska, Markus Fischer, Jan Fritsche

**Affiliations:** 1 Department of Safety and Quality of Milk and Fish Products, Max Rubner-Institut (MRI), Hermann-Weigmann-Str. 1, 24103 Kiel, Germany; 2 Present Address: National Reference Centre for Authentic Food, Max Rubner-Institut (MRI), Hermann-Weigmann-Str. 1, 24103 Kiel, Germany; 3 Institute of Food Chemistry - Hamburg School of Food Science, University of Hamburg, Grindelallee 117, 20146 Hamburg, Germany

**Keywords:** Authentication, Crustaceans, DNA microarray, Giant tiger prawn, Shrimp species, Whiteleg shrimp

## Abstract

This proof-of-principle study describes the development of a rapid and easy-to-use DNA microarray assay for the authentication of giant tiger prawns and whiteleg shrimp. Following DNA extraction and conventional end-point PCR of a 16S rDNA segment, the PCR products are hybridised to species-specific oligonucleotide probes on DNA microarrays located at the bottom of centrifuge tubes (ArrayTubes) and the resulting signal patterns are compared to those of reference specimens. A total of 21 species-specific probes were designed and signal patterns were recorded for 47 crustacean specimens belonging to 16 species of seven families. A hierarchical clustering of the signal patterns demonstrated the specificity of the DNA microarray for the two target species. The DNA microarray can easily be expanded to other important crustaceans. As the complete assay can be performed within half a day and does not require taxonomic expertise, it represents a rapid and simple alternative to tedious DNA barcoding and could be used by crustacean trading companies as well as food control authorities for authentication of crustacean commodities.

**Supplementary Information:**

The online version contains supplementary material available at 10.1007/s00216-021-03440-2.

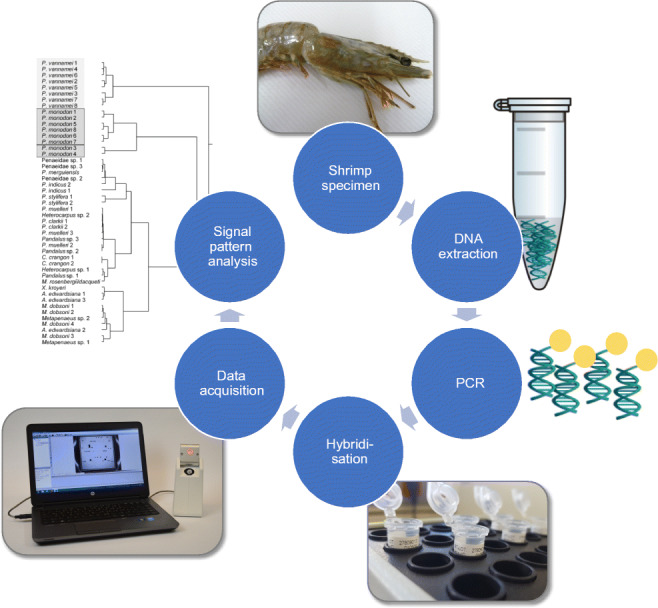

## Introduction

Crustaceans such as shrimp and prawns are popular food commodities that are traded worldwide. Two of the most important species are the giant tiger prawn (*Penaeus monodon*) and the whiteleg shrimp (*Penaeus* (*Litopenaeus*) *vannamei*). Global production (capture and aquaculture) in 2018 was 4.98 million tons for *P. vannamei* and 0.98 million tons for *P. monodon*, respectively (data taken from http://www.fao.org/fishery/statistics/global-production/query/en). This represented more than half of the total amount of produced shrimp and prawns in that year.

In many countries, such as European Union (EU) member states, importers, manufacturers and retailers are required to label fishery products with the scientific name, unless the products are prepared with other ingredients (dictated by Regulation (EU) No 1379/2013 on the common organisation of the markets in fishery and aquaculture products). Since shrimp and prawn products are often exported and traded as processed products (e.g. peeled and cooked specimens), it is usually not possible to verify whether the purchased products are authentic in relation to the declared species without elaborate laboratory analyses. Therefore, species-level crustacean authentication methods are needed for both food control and food industry (or respective service laboratories) to verify and ensure proper labelling of traded crustacean products. The usual procedure for the authentication of crustacean products is to amplify a specific DNA fragment by PCR and sequence the resulting amplicons. The determined sequences have to be compared with DNA sequences in public databases such as GenBank [1] and BOLD [2]. However, this DNA barcoding not only is time consuming and tedious, but also requires considerable expertise in the taxon under study as well as the quality of the sequence data in the databases. Ambiguous and conflicting sequences in the public databases can complicate the interpretation of query results, as was recently described for snapper species, for example [3]. Thus, commissioning service laboratories to authenticate species authentication for self-monitoring analyses is quite expensive and the processing time usually takes several days.

As an alternative to DNA barcoding-based species authentication, species-specific methods such as real-time PCR assays or hybridisation-based assays using DNA microarrays (DNA chips, biochips) could be used. These methods offer the advantage that the complete sequence data in the public databases have already been assessed when the method was developed and do not need to be evaluated each time the method is applied. Therefore, they can be used by technical personnel without requiring scientific expertise in taxonomy. DNA microarray-based assays are based on binding amplified target DNA from the sample to immobilised oligonucleotide probes bound to the microarray surface. When designing the oligonucleotide probe sets, all available sequence information can be reviewed and assessed for correctness. Commercially available DNA chips are already being used to differentiate animal species in meat products [4]. Because of the reduced variety of animal species expected in meat products, the oligonucleotide probes on “meat chips” do not have to be able to differentiate between closely related species. Thus, a one-probe-per-species approach is usually sufficient for a valid species authentication. However, crustacean species are much more diverse, and genetically related species are often used as substitutes in seafood commodities; therefore, one probe per species is usually not enough for identifying crustacean species with DNA microarrays.

Recently, it was shown that it is possible to authenticate closely related fish species using a sophisticated probe design and signal pattern analysis [5]. Therefore, the aim of the present study was to test whether two crustacean species, the giant tiger prawn (*P. monodon*) and the whiteleg shrimp (*P. vannamei*), can be specifically detected using a DNA microarray assay followed by a hierarchical clustering comparison of probe signal patterns. It should provide initial evidence that DNA chips are useful tools for authenticating crustacean food commodities. These DNA microarrays could be used in simple and rapid procedures by both food inspection agencies and service laboratories that perform authenticity tests on imported or purchased goods on behalf of companies marketing crustacean products.

## Materials and methods

### Statement of human and animal rights

No experiments with humans or live animals were conducted.

### Oligonucleotide probe design and DNA microarray fabrication

Oligonucleotide probes (Table 1) were designed to bind to an approximately 310-bp 16S rDNA PCR amplicon (see below). All 16S rDNA sequences of the target species *Penaeus* (*Litopenaeus*) *vannamei* and *Penaeus monodon* as well as DNA sequences of related shrimp and prawn species (*Penaeus* (*Litopenaeus*) *stylirostris*, *Penaeus* (*Litopenaeus*) *setiferus*, *Penaeus* (*Fenneropenaeus*) *indicus*, *Penaeus* (*Marsupenaeus*) *japonicus*, *Penaeus semisulcatus*, *Metapenaeus affinis*) were downloaded from GenBank in January 2017 and aligned with Clustal X 2.1 [6]. At that time, 40 sequences were identified for *P. vannamei* of which 32 sequences represented the same 16S rDNA segment compared to the chosen fragment for this study. *P. monodon* exhibited 72 16S rDNA sequences in GenBank and most of the sequences could be divided into two main clusters. For the probe design, the most frequent haplotypes of all species were compiled in one alignment. Species-specific probe sequences of 20 nucleotide lengths with as many nucleotide differences to related species as possible (preferably located in the probe centre) were visually identified for *P. vannamei* and *P. monodon*. The melting temperatures of the probes (T_M_) were calculated with Oligonucleotide Properties Calculator (Oligo Calc) [7] using a nearest neighbour model, and the probes were adjusted to feature similar melting temperatures by elongation or truncation. Two *P. monodon* probes were designed in two types each, to match the sequences in the two different *P. monodon* 16S rDNA clusters. Additional mismatch (MM) probes were designed, which represented probe variations with nucleotide positions complementary to related non-target species in order to achieve a better differentiation from species with highly similar 16S rDNA sequences. A universal probe, originally designed for the detection of 16S rDNA targets from fish DNA (already described in 6), was selected as an internal positive control. Three probes without complementarity to crustacean 16S rDNA sequences were used as negative control probes (also from 6). All oligonucleotides probes were synthesized by Metabion International AG (Planegg, Germany) with a terminal C7 amino link at the 3’ end. Special care was taken to avoid biotin contaminations.

**Table 1:** Oligonucleotide probes

Target species	Probe name	Probe sequence (5' -> 3')a	Length (nt)	GC content (%)	T_M_ (° C)
Internal positive control	uni_16S_05b	TTACGACCTCGATGTTGG	18	50	48.4
*P. vannamei*	Pvannam_16S_01	TGTCTCAATTATATTTATTGAATTTA	26	15	46.6
*P. vannamei*	Pvannam_16S_02	TTACAATAAGTTACCTATATTATAAA	26	15	45.1
*P. vannamei*	Pvannam_16S_02_MM	TTACAATAAGTTAtCTATATTATAAA	26	12	43.7
*P. vannamei*	Pvannam_16S_03	GAGTTTAGGTAACGTTTGTT	20	35	45.5
*P. vannamei*	Pvannam_16S_04	GTTCTTAAGTTATTTAATGACAG	23	26	45.5
*P. vannamei*	Pvannam_16S_04_2MM	GTTCTTAAGcTATTTAATaACAG	23	26	46.2
*P. vannamei*	Pvannam_16S_04_3MM	GTTCTTAAGTTAaTTAATaACAt	23	17	42.3
*P. vannamei*	Pvannam_16S_05	AATGACAGAAATTTCTGGAAA	21	29	46.4
*P. vannamei*	Pvannam_16S_06	GATCCTCTACTAGAGATCA	19	42	45.0
*P. vannamei*	Pvannam_16S_06_2MM	GATCCTCTttTAGAGATCA	19	37	44.0
*P. monodon*	Pmonod_16S_01	CAAAAAGTAATCTGTCTCAG	20	35	45.4
*P. monodon*	Pmonod_16S_01_MM	CAAAAAGTAAgCTGTCTCAG	20	40	48.4
*P. monodon*	Pmonod_16S_02	GCTTAAATACTTTAAGGGGA	20	35	45.3
*P. monodon*	Pmonod_16S_03ac	ACAATAATTTGATTAAATTATAAATT	26	8	43.6
*P. monodon*	Pmonod_16S_03bc	ACAATAAATTAGTTAAATTATAAATT	26	8	43.2
*P. monodon*	Pmonod_16S_04	GGAATATAATTAGTAACTGTTC	22	27	43.0
*P. monodon*	Pmonod_16S_04_MMa	GGAATATAATaAGTAACTGTTC	22	27	43.0
*P. monodon*	Pmonod_16S_04_MMb	GGAATATAATgAGTAACTGTTC	22	32	45.1
*P. monodon*	Pmonod_16S_05ac	AGTATAATTGAAGAATAATTGATC	24	21	45.3
*P. monodon*	Pmonod_16S_05bc	AGTATAATTGAAAAATAATTGATC	24	17	43.8
*P. monodon*	Pmonod_16S_06	ATTGATCCTTTATTAAAGATTAA	23	17	43.9
Negative control	NK01 ^b^	TACCAACTTCGCTAACTCA	19	42	48.5
Negative control	NK02 ^b^	ATATTCTGCCCGCAGTTA	18	44	48.2
Negative control	NK03 ^b^	TTGTGCCATTCTTGAAAGATC	21	38	49.6

^a^Lowercase letters indicate mismatch (MM) positions (i.e. positions with bases complementary to related species)

^b^Published in Kappel et al. (2020) [6]

^c^“a” and “b” represent two types of probe, each for a distinct *P. monodon* 16S rDNA cluster

The oligonucleotide probes were spotted in triplicates onto ArrayTube2 DNA microarrays by Alere Technologies (Jena, Germany) in concentrations of 15 μM. An additional biotin labelled nucleotide, selected by the manufacturer, was spotted in order to serve as a control for the staining reaction.

### Collection of test specimens and species assignment

#### Test specimens

To verify the developed method in terms of specificity, specimens from as many shrimp species as possible (see Table 2 and Supplementary Information (ESM) Table S1) were obtained as seafood products from local producers or retailers, had been collected in previous projects or were bought at a local wholesale and retail shop. First of all, the species of all specimens were determined based on PCR and sequencing of an approximately 312-bp fragment of the mitochondrial 16S rDNA according to the official method for species identification of crustaceans in Germany [8]. A subset of samples was additionally analysed by sequencing a longer 16S rDNA fragment [9] and/or a part of the barcoding gene COI [10] in order to obtain a more reliable species assignment.

**Table 2:** Crustacean specimens used in this study. The target species are indicated in bold

Order	Suborder	Superfamily	Family	Species	Analysed specimens^a^
**Decapoda**	**Dendrobranchiata**	**Penaeoidea**	**Penaeidae**	***Penaeus*** **(*Litopenaeus*)** ***vannamei***	**8**
**Decapoda**	**Dendrobranchiata**	**Penaeoidea**	**Penaeidae**	***Penaeus monodon***	**8**
Decapoda	Dendrobranchiata	Penaeoidea	Penaeidae	*Penaeidae* sp.	3
Decapoda	Dendrobranchiata	Penaeoidea	Penaeidae	*Penaeus* (*Fenneropenaeus*) *indicus*	2
Decapoda	Dendrobranchiata	Penaeoidea	Penaeidae	*Penaeus* (*Fenneropenaeus*) *merguiensis*	1
Decapoda	Dendrobranchiata	Penaeoidea	Penaeidae	*Parapenaeus stylifera*	2
Decapoda	Dendrobranchiata	Penaeoidea	Penaeidae	*Xiphopenaeus kroyeri*	1
Decapoda	Dendrobranchiata	Penaeoidea	Penaeidae	*Metapenaeus dobsoni*	4
Decapoda	Dendrobranchiata	Penaeoidea	Penaeidae	*Metapenaeus* sp.	2
Decapoda	Dendrobranchiata	Penaeoidea	Solenoceridae	*Pleoticus muelleri*	3
Decapoda	Dendrobranchiata	Penaeoidea	Aristaeidae	*Aristaeopsis edwardsiana*	3
Decapoda	Pleocyemata	Crangonoidea	Crangonidae	*Crangon crangon*	2
Decapoda	Pleocyemata	Pandaloidea	Pandalidae	*Heterocarpus* sp.	2
Decapoda	Pleocyemata	Pandaloidea	Pandalidae	*Pandalus* sp.	3
Decapoda	Pleocyemata	Palaemonoidea	Palaemonidae	*Macrobrachium rosenbergii*/*dacqueti*	1
Decapoda	Pleocyemata	Astacoidea	Cambaridae	*Procambarus clarkii*	2
				Sum:	47

^a^Detailed results for BLAST and BOLD queries for all specimens can be viewed in ESM Table S1

#### DNA extraction

Total DNA was extracted using different methods. The DNeasy Blood & Tissue Kit (Qiagen, Hilden, Germany) and the E. Z. N. A. Tissue DNA Kit (Omega Bio-tek, Norcross, GA, USA) were applied according to manufacturers’ instructions. A CTAB extraction protocol was also used according to Rehbein [11]. In brief, a small piece of muscle tissue was dissected from a specimen and incubated with 500 μl extraction buffer (buffer 1) (1.2% CTAB (w/v), 60 mM Tris, 10 mM Na_2_-EDTA, 0.8 M NaCl; pH 8.0) in the presence of 12.5 μl Proteinase K (20 mg/ml) and 0.5 μl 3-mercapto-1,2-propanediol for 1 to 2 h at 65 °C and 300 rpm in a heat block. After centrifugation (10 min, 13,000 rpm), the supernatant was transferred into a microcentrifuge tube and was gently mixed with one volume chloroform. The solution was centrifuged again and the supernatant was transferred into a new tube. The chloroform extraction step was repeated once. Afterwards, two volumes of buffer 2 (1% CTAB, 50 mM Tris, 10 mM Na_2_-EDTA; pH 8.0) were added to the supernatant, the solution was swiveled gently, incubated for 5 min at room temperature and centrifuged again (10 min, 13,000 rpm). After discarding the supernatant, the precipitate was resolved in 400 μl buffer 3 (1 M NaCl, 10 mM Tris, 1 mM Na_2_-EDTA; pH 8.0) and was subsequently incubated for 10 min at 65 °C. The DNA was precipitated with one volume isopropyl alcohol for 10 min at room temperature, centrifuged for 10 min at 13,000 rpm; the supernatant was discarded and the precipitate was washed twice with 500 μl ethanol (70%). Finally, the DNA precipitate was air dried and afterwards dissolved in 100 μl buffer 4 (10 mM Tris, 1 mM Na_2_-EDTA; pH 8.0).

DNA concentrations and purities were measured in a microvolume spectrophotometer. The concentrations were adjusted to 10 ng/μl with buffer 4 and the DNA solutions were stored at −20 °C. Within each DNA extraction session, a negative control was performed as a last sample (starting with dipping the scalpel and forceps into the extraction buffer). This extraction control was analysed once with PCR (see below) in order to check for cross-contaminations during the extraction sessions.

#### 16S rDNA and COI PCRs

All primers for PCR were synthesized by biomers.net (Ulm, Germany). An approximately 310-bp 16S rDNA fragment (hereinafter referred to as short 16S rDNA fragment) was amplified with the primers 16S 312F (5’-ccagggttttcccagtcacgGRAGGCTTGTATGAATGGTTG-3’) and 16S 312R-1 (5’-cggataacaatttcacacaggAARWARATWACGCTGTTA-3’) [8, 12]. The lowercase letters indicate the M13-tails as binding regions for the sequencing primers. The PCRs were performed in final volumes of 20 μl and contained 10 μl AccuStart II PCR Supermix (Quantabio, Beverly, MA, USA), 500 nM of each primer and 20 ng extracted DNA. The PCR conditions were as follows: 3 min initiate denaturation at 94 °C, followed by 35 cycles with 30 s at 94 °C denaturation, 30 s annealing at 50 °C and 60 s elongation at 72 °C.

A subset of specimens was also analysed by sequencing a longer 16S rDNA fragment of approximately 520 bp (hereinafter referred to as long 16S rDNA fragment). The primers have been described by Palumbi et al. [9] and were elongated with M13-tails for sequencing: 16sar-L (5’-ccagggttttcccagtcacgCGCCTGTTTATCAAAAACAT-3’) and 16sbr-H (5’-cggataacaatttcacacaggCCGGTCTGAACTCAGATCACGT-3’). The reaction compositions were as above and the reaction scheme was as follows: 3 min initial denaturation at 95 °C, 35 cycles with 30 s denaturation at 94 °C, 30 s annealing at 50 °C and 45 °C elongation at 72 °C, and a final elongation step for 10 min at 72 °C.

For some specimens, COI was additionally determined according to a modified protocol by Lobo et al. [10] using the primers LoboF1 (5’-ccagggttttcccagtcacgKBTCHACAAAYCAYAARGAYATHGG-3’) and LoboR1 (5’-cggataacaatttcacacaggTAAACYTCWGGRTGWCCRAARAAYCA-3’) (lowercase letters indicating M13-tails) and the following PCR programme: initial denaturation for 3 min at 94 °C, five cycles with 30 s at 94 °C, 90 s at 45 °C and 60 s at 72 °C, another 40 cycles with 30 s at 94 °C, 90 s at 54 °C and 60 s at 72 °C, and a final elongation for 5 min at 72 °C.

A no template control (NTC) was included in each reaction in order to check for cross-contaminations during the preparation of the reactions.

#### Sequencing and sequence editing

The amplicons were checked on 2% agarose gels. If the electrophoresis results were satisfactory, amplicons were diluted one to ten in molecular biology grade water (without further purification) and were sent to LGC Genomics (Berlin, Germany) for being sequenced in both directions using the M13-24F and M13-24R sequencing primers. Upon arrival of the sequencing results, the sequences were edited in Chromas Lite 2.6.6 (Technelysium Pty Ltd, South Brisbane, Queensland, Australia) by inspecting the electropherogram raw data. The primer sequences were removed from the sequences, the correctness of the base calling was checked and the bases were corrected when necessary. Forward and reverse sequences were assembled.

#### BLAST and BOLD queries

The 16S rDNA sequences as well as the COI sequences were compared to GenBank sequences by BLASTn (https://blast.ncbi.nlm.nih.gov/Blast.cgi). The maximum number of hits was set to 5000 and the resulting hits were sorted by maximum percent identity. In cases, where the hits with the highest identities differed from the hits with the highest maximum score, the first hits according to the latter score were also displayed. The COI sequences were also queried in BOLD using the Species Level Barcode Records. A compilation of the BLASTn and BOLD query results can be found in ESM (Table S1).

Species assignment was made, in cases where the query results obtained were unambiguous, i.e. multiple hits displayed sequence identities of at least 99% to a given species, query coverage was 100% or only slightly less and the hits with high identities (≥ 98%) were annotated with only one species. In cases where hits with high identities were annotated with multiple species, assignment was made only to genus or family level. If only individual records with high identities differed from the other hits with respect to the annotated species, these hits were considered misidentified specimens and ignored. If different specimens where assigned to a particular species but showed significant sequence discrepancy among each other, p-distances were calculated with MEGA7 [13] for discussion of the results.

### DNA microarray hybridisation experiments

#### Preparation of target DNA

The target DNA for the microarray experiments was prepared from the DNA extracts of the test specimens by amplifying the long 16S rDNA fragment (see above) in a conventional PCR utilising a 5’ biotin-labelled reverse primer. The presence of amplicons was checked with gel electrophoresis using 2% agarose gels. The amplicons were hybridised without prior purification or photometric measurement in a final dilution of 1:500 on the DNA microarrays (see below).

#### DNA microarray hybridisation

The microarray hybridisation experiments were performed using the hybridisation kit appropriate for the ArrayTubes (Alere Technologies) according to the manufacturer’s recommendations. First, the DNA targets (16S rDNA amplicons) were diluted 1:50 with water and denatured for 5 min at 95 °C. Immediately thereafter, they were placed on ice and kept there until hybridisation. The DNA microarrays in the ArrayTubes were rinsed briefly with water and were pre-incubated with 200 μl prehybridisation solution for 2 min at 48 °C. Afterwards, 10 μl of the denatured amplicons was mixed with 90 μl of cold buffer C1 and the mixture was pipetted onto the DNA microarrays and incubated for 1 h at 48 °C under gentle agitation (550 rpm). After hybridisation, the microarrays were washed three times with buffer C2 before incubation with streptavidin-horseradish conjugate for 10 min at 30 °C. After two more washing steps, the microarrays were stained with 100 μl of staining solution D1 for 5 min at 30 °C. They were measured directly in the ArrayTube Reader (ATR03) and evaluated using the Iconoclust software supplied by the manufacturer (both Alere Technologies).

#### DNA microarray data evaluation

The hybridisation signals were recorded in arbitrary units (a.u.) and were further processed in Microsoft Excel 2010 (Microsoft, Redmon, WA, USA). Mean signal values and standard deviations were calculated from probe triplicates. If the signal from one probe spot deviated significantly from the other two spots of the particular probe, the deviating spot was excluded from the analysis.

In order to verify that the signal patterns were specific for *P. monodon* and *P. vannamei*, the signal patterns of all investigated specimens from target as well as non-target species were compared with a hierarchical clustering approach in JMP 13.2.1 (SAS Institute, Cary, NC, USA) with the Ward method without standardisation.

## Results and discussion

### Identification of crustacean specimens at species level

First, the samples had to be authenticated to species level using conventional PCR and sequencing methods before they could be used as test samples for verification of the DNA microarray approach. However, a clear assignment to a specific species was not possible in all cases (see below). The results of the BLAST and BOLD searches are summarised in ESM (Table S1). Since the short 16S rDNA fragment is completely included in the long 16S rDNA fragment, only the results for the long fragment are listed in cases where the long fragment was analysed in addition to the short fragment. Table 2 shows the compilation of samples with the annotation of the identified species.

#### Target species *P. monodon* and *P. vannamei*

Eight individuals from three whiteleg shrimp products with three different declared origins (Indonesia, Vietnam and Ecuador) and one crustacean product without exact origin specification could be unambiguously assigned to *Penaeus* (*Litopenaeus*) *vannamei* (see Table 2 and ESM Table S1). The 16S rDNA sequences were identical among the eight specimens and showed 100% identity to *P. vannamei* GenBank entries. One specimen was additionally analysed with COI, which also resulted in GenBank as well as BOLD hits with 100% identity.

Eight samples from four giant tiger prawn products (from Vietnam, India and Bangladesh and one specimen without origin declaration) could be clearly assigned to *Penaeus monodon*. However, the sequences of two specimens (from Vietnam) differed significantly from the sequences of the other *P. monodon* specimens. The mean p-distance between the two groups was 0.017 for the long 16S rDNA sequences, and the p-distance between the two analysed COI sequences (one from each group) was 0.07. Especially the latter p-distance is quite uncommon for specimens belonging to the same species. Considerable genetic diversity of *P. monodon* has been described by many authors (e.g. [14–16]) and may be due to different geographic origins or even indicate the presence of cryptic species within the taxon *P. monodon* [17]. Nevertheless, both groups appear to be marketed as giant tiger prawns and shall be considered as such for this study, as resolution of taxonomy is beyond the scope of this study. The genetic diversity of *P. monodon* was considered in the probe design, and two probes were defined in two variants each (one probe variant per group) (see above).

#### Other Penaeidae species

Four shrimp specimens from three shrimp products were identified as *Metapenaeus dobsoni* (Kadal shrimp) according to the GenBank BLAST and BOLD results for the COI sequences of two of the four specimens (see ESM Table S1) and the similarity of the 16S rDNA sequences between all four specimens. However, the p-distance between the COI sequences of both analysed specimens was 0.047, which is usually too high for belonging to the same species. This corresponds to the sequence divergence of the 50 *M. dobsoni* COI entries identified in GenBank that form two separate groups when compared on sequence level. One of the specimens from this study grouped with one *M. dobsoni* cluster (with 40 sequence entries) and the other specimen with another *M. dobsoni* cluster (with seven sequence entries). Possible explanations for this might be the presence of cryptic species within the taxon *M. dobsoni* or falsely annotated sequences belonging to a species with no COI sequences as well as no 16S rDNA sequences already existing in GenBank, but a clarification of this situation was outside the scope of this study. It is also noteworthy that the only 16S rDNA sequence for *M. dobsoni* in GenBank displayed only 94.4% identity to the sequences of the four specimens from this study, which was less than the maximum identities found in the BLAST search results, which were 95.39% and 96.78% to *M. tenuipes*.

One specimen was assigned to *Xiphopenaeus kroyeri* (Atlantic seabob) according to the short 16S rDNA sequence as well as the COI sequence. However, again, the 16S rDNA query sequence and the COI query sequence matched the database sequences of *X. kroyeri* not only with 100% and 99.5 to 99.6% identity, respectively, but also with much lower identities (95.96 to 96.69% and less than 90%, respectively), which again could indicate the presence of cryptic species, as recently described for the Atlantic seabob [18].

Two individuals from a wild caught shrimp product (caught in the Western and Eastern Indian Ocean and the middle West Pacific) were likely to be *Parapenaeopsis stylifera* (kiddi shrimp) based on 16S rDNA (long fragment) and COI, although there were only few 100% matches in GenBank and BOLD.

Three specimens declared as *Penaeus* (*Farfantepenaeus*) *notialis* (southern pink shrimp) could not be verified as such because they exhibited only 96.32% (short 16S rDNA fragment) and 97.07% (COI) identity to *P. notialis* sequences from GenBank and 97.06% similarity to BOLD sequences. As no hits could be obtained with sufficiently high identities to any species, the assignment was made only to family level (“Penaeidae sp.”)

Two specimens were assigned to *Penaeus* (*Fenneropenaeus*) *indicus* (Indian white prawn), but again, the database entries featured sequences with extremely high diversities indicating the presence of cryptic species (see also [19]). Both individuals from the present study seem to represent specimens from the two different cryptic species as the identity between the COI sequences was only 86.8%.

Another specimen was likely to be *Penaeus* (*Fenneropenaeus*) *merguiensis* (banana prawn), based on the database results for the long 16S rDNA fragment and COI. However, the sequences of this specimen were more similar to the sequences from one of the two *P. indicus* specimens than the sequences of those two specimens were similar among each other.

Two specimens could only be assigned at the genus level, namely to *Metapenaeus* sp., because the 16S rDNA and COI sequences of several *Metapenaeus* species (such as *M. affinis*, *M. ensis*, *M. monoceros*) were too similar between species on the one hand, but too divergent within species on the other.

#### Additional shrimp and prawn species

Two specimens could be clearly assigned to *Crangon crangon *(common shrimp) and three other specimens to *Pleoticus muelleri* (Argentine red shrimp). Three specimens obtained as *Penaeus edwardsianus* (unaccepted name) were most likely to be *Aristaeopsis edwardsiana* (scarlet shrimp), although there are only very few sequences in the public databases: in GenBank, there are two 16S rDNA sequences but no COI sequences for *A. edwardsiana*, and BOLD features only two public records for this species.

Wild Chilean nylon shrimp (*Heterocarpus reedi*) were obtained from a local supermarket but could only be verified to genus level (*Heterocarpus* sp.), as data for this species were not present either in GenBank nor in BOLD. Another Pandalidae species was obtained as *Pandalus borealis* (northern shrimp), but could only be assigned to genus level (*Pandalus* sp.) due to the similarity of *P. borealis* and *P. eous* (Alaskan pink shrimp) sequences in GenBank and BOLD.

A giant river prawn specimen (*Macrobrachium rosenbergii*) could be verified as such, although information in the public databases again diverged. This could be explained by the findings of Wowor and Ng [20], who reported that the originally described *M. rosenbergii* species consist of two distinct species, *M. rosenbergii* (de Man, 1879) and *M. dacqueti* (Sunier, 1925). Since the latter species is one of the most commercially important crustaceans of the world [20], the specimen from this study could also be *M. dacqueti*, for which sequences are not yet available in GenBank.

#### Crayfish specimens

In addition to the shrimp samples, two crayfish specimens were obtained as a cooked product (origin: Chinese inland fisheries) and identified as *Procambarus clarkii* (red swamp crayfish) based on a solid data basis in GenBank and BOLD.

#### Need for improvement of crustacean sequence data

Although it was not within the scope of this study to assess the completeness of the sequence data in the public databases GenBank and BOLD, it nevertheless became clear that the data situation for crustacean species in the public sequence repositories urgently needs to be improved. Otherwise, both the competent food control authorities and the respective private laboratories will not be able to verify the authenticity of crustacean products that have been or will be placed on the market. Therefore, projects aimed at improving and completing the sequence data of crustacean species available on the international markets should be encouraged. Such projects should preferably be multinational projects carried out primarily by countries where the species are caught or harvested, since only these countries have the rights to use the genetic resources without prior authorisation under the Nagoya Protocol on Access and Benefit Sharing (ABS) (see https://www.cbd.int/abs).

### DNA microarray probe signal patterns

**Fig. 1:**
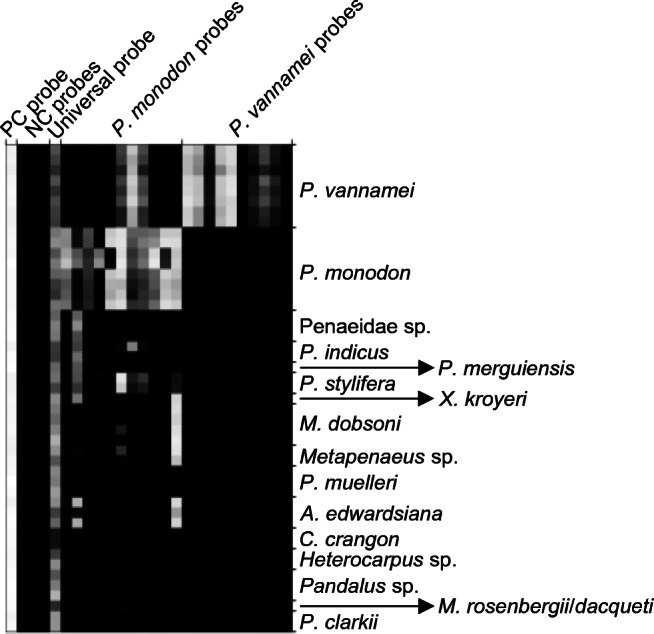
Visualisation of the DNA microarray probe signal patterns for all investigated target and non-target specimens as a heat map. The different probes are arranged in the columns; the analysed specimens in the rows. The probe signals range from 0 a.u. (black) to 0.8 a.u. (white). The heat map was produced with Microsoft Excel 2010 (Microsoft, Redmond, WA, USA)

**Fig. 2:**
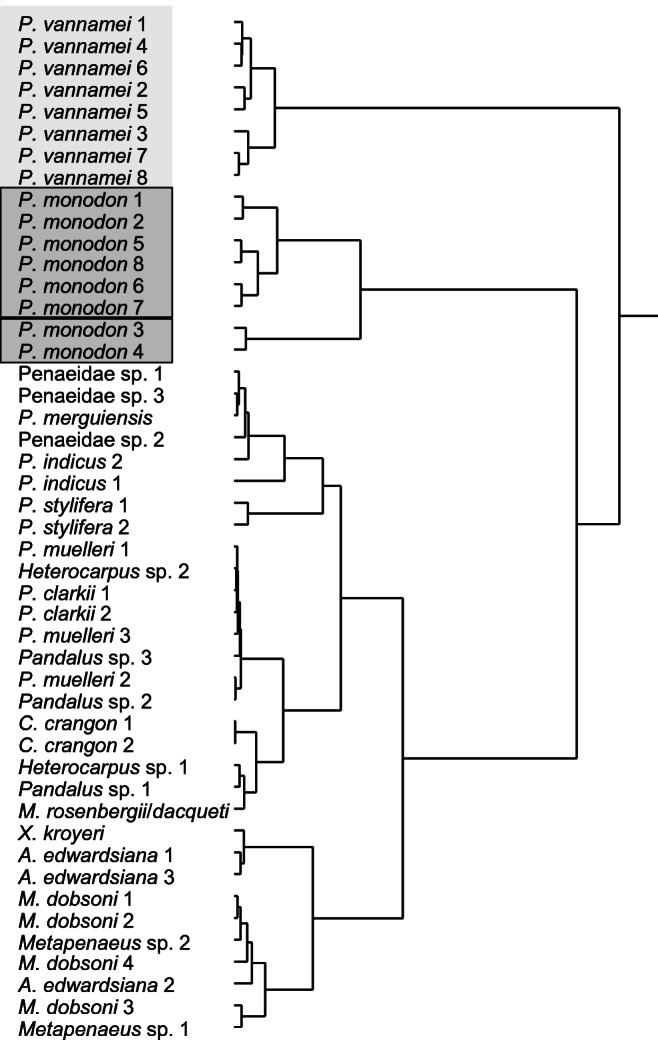
Hierarchical clustering dendrogram of probe signal patterns for all investigated target (highlighted in grey) and non-target test specimens. For the clustering, the Ward method was used without standardization. The two different *P. monodon* groups which might represent geographically distinct populations or even cryptic species are indicated in boxes

The ArrayTube DNA microarrays containing a total of 25 oligonucleotide probes, each in triplicate, were tested in separate hybridisation experiments with amplicons from eight specimens each of the target species *P. vannamei* and *P. monodon*, 15 specimens from seven other Penaeidae species and 16 specimens from seven additional species belonging to six different families (see Table 2 and Fig. 1; the raw data of the signal intensities can be viewed in ESM Table S2). The staining control probe yielded uniformly high signals (mean: 0.82 a.u.; standard deviation: 0.008 a.u.). The universal probe reacted with all analysed specimens; however, the signal levels could not be compared and ranged from very low (0.18 a.u.) to very high (0.7 a.u.). All negative control probes did not react with any of the amplicons and showed signal intensities compared to background level (max: 0.024 a.u.; mean: 0.003 a.u.). All probes designed to match to *P. vannamei* sequences did not show cross-hybridisations with any other species. Two probes (Pmonod_16S_04 and Pmonod_16S_06) designed for the detection of *P. monodon* hybridised also with other species as did three of the designed mismatch probes, as expected. One *P. monodon* probe and the two corresponding mismatch probes showed also signals with *P. vannamei* amplicons. However, the overall patterns of the probe signals proved specific for the two target species, as can be seen in the dendrogram of the hierarchical clustering approach (Fig. 2). The target species (highlighted in grey) could be clearly distinguished from each other and from all other species studied. With the proposed probe set, it was even possible to distinguish the specimens belonging to the two genetically distinct *P. monodon* groups (highlighted by rectangles) which probably represent geographically differentiated populations or cryptic species (see also above).

The complete experimental setup of the DNA microarray-based species authentication comprised standard labware of a PCR laboratory including a microcentrifuge, a microvolume spectrophotometer for measuring the DNA concentration, a PCR cycler for target amplification and a conventional heat block preferably with additional cooling function to speed up the hybridisation and washing steps. The only additional device needed was the ArrayTube reader connected to a computer or notebook for data acquisition of the probe signals. The overall procedure excluding the DNA extraction could be accomplished within 3.5 h. With a rapid DNA extraction protocol, the whole procedure will take less than 4 h. However, the main advantage is that the complete assay can be performed by technical personnel alone. First of all, the probe signal patterns of authentic reference specimens have to be collected. These can be saved in digital format. The signal pattern of a test specimen can then be compared to the reference patterns in order to decide whether the specimen belongs to the species in question (in this case *P. monodon* or *P. vannamei*) or not. The evaluation of the frequently confusing sequences in the public databases has already been done beforehand.

It has to be considered that each probe that proved specific in the setup of this study is very likely to bind to any other species due to the vast number of crustacean species occurring worldwide. In addition, there are likely to be giant tiger prawn and whiteleg shrimp specimens that have point mutations at probe binding positions that may result in false-negative probe signals. This is a problem inherent to all nucleic-based species-specific methods. The proposed DNA microarray-based approach circumvents this problem by using a complete probe set instead of a single probe per species. The chance that the complete probe set for giant tiger prawn or whiteleg shrimp will bind to the DNA of another non-target species or fail on a specific sample is very low. In this regard, DNA microarray-based species authentication has an advantage over, for example, real-time PCR-based species authentication methods.

## Conclusion

This study has shown that DNA microarrays are a promising alternative to sequencing-based approaches for authentication of crustacean products with respect to the declared species. Despite the vast number of crustacean species on the global food market and the possibility of substitution with closely related species, certain species can be specifically identified with oligonucleotide probe sets comprising several probes per species. Instead of relying on one probe per species, the overall probe signal patterns are compared to the digital signatures of those of authentic reference specimens using a hierarchical clustering algorithm. This approach makes the species authentication much more robust against false-positive assignments (when otherwise a closely related species would also hybridise to the probe in question) and false-negative assignments (when otherwise a specimen with a mutation at the probe binding position would fail to bind to the probe).

The entire procedure can be applied in a molecular biology laboratory without requiring a sequencing instrument but only standard equipment plus an additional ArrayTube reader. Thus, laboratories without sequencing facilities can perform the authentication of crustacean specimens without subcontracting a sequencing service provider, and, with a fast DNA extraction protocol, the whole procedure only takes half a day compared to several days for a sequencing order. Because DNA microarrays can have hundreds to thousands or, depending on the microarray platform chosen, even more probe binding positions, they allow the development of test systems that can be used for many species. Thus, the next step would be to extend the oligonucleotide probe set to other crustacean species relevant for the market. In this context, this study represents a proof-of-concept for DNA microarrays as easy-to-use and rapid tools for species authentication of crustacean commodities for self-monitoring of purchased goods by crustacean trading companies, as well as for market controls by the relevant food inspections authorities. Further developments should include automation of the assay using microfluidic chips, to enable in-house testing of crustacean samples for manufacturers, distributors and retailers in the future.

## Supplementary Information

ESM 1 (PDF 238 kb)

ESM 2 (XLSX 27 kb)
