# Supplementary material for: A DNA microarray for the authentication of giant tiger prawn (Penaeus monodon) and whiteleg shrimp (Penaeus (Litopenaeus) vannamei): a proof-of-principle
Source: Anal Bioanal Chem. 2021 Jun 11;413(19):4837–46. doi: 10.1007/s00216-021-03440-2 (PMC8318932; doi:10.1007/s00216-021-03440-2)
Supplement: Supplementary file 1 — (PDF 238 kb) [file 216_2021_3440_MOESM1_ESM.pdf]

Supplementary Information

A DNA microarray for the authentication of giant tiger prawn (*Penaeus monodon*) and whiteleg shrimp (*Penaeus* (*Litopenaeus*) *vannamei*): a proof-of-principle

Analytical and Bioanalytical Chemistry

Kristina Kappel, Joanna Fafińska, Markus Fischer, Jan Fritsche

Corresponding author: Kristina Kappel, Max Rubner-Institut, Department of Safety and Quality of Milk and Fish Products, Kiel, Germany; kristina.kappel@mri.bund.de

Table S1 In this file, the BLAST and BOLD results from database queries with sequences obtained from the crustacean specimens used in this study are compiled

Species	Specimen	Original sample designation	Gene marker (and database)	First GenBank hit (sorted by Maximum Percent Identity/sorted by Maximum Score)[a] /First BOLD hit	GenBank identity of first hit (sorted by Maximum Percent Identity/sorted by Maximum Score)[a] / BOLD similarity of first hit
**Penaeidae**					
Penaeus* (*Litopenaeus*) *vannamei	*P. vannamei* 1	Pvan01a	16S rDNA (long)	Litopenaeus vannamei (KT596762)	100% (519 nt/519 nt)
			COI GenBank	Penaeus vannamei (MH194528)	100% (658 nt/658 nt)
			COI BOLD	Penaeus vannamei;<br>Litopenaeus vannamei	100%
	P. vannamei 2	Pvan01b	16S rDNA (long)	Litopenaeus vannamei (KT596762)	100% (519 nt/519 nt)
	P. vannamei 3	Pvan02a	16S rDNA (long)	Litopenaeus vannamei (KT596762)	100% (519 nt/519 nt)
	P. vannamei 4	Pvan02b	16S rDNA (long)	Litopenaeus vannamei (KT596762)	100% (519 nt/519 nt)
	P. vannamei 5	Pvan03a	16S rDNA (long)	Litopenaeus vannamei (KT596762)	100% (519 nt/519 nt)
	P. vannamei 6	Pvan03b	16S rDNA (long)	Litopenaeus vannamei (KT596762)	100% (519 nt/519 nt)
	P. vannamei 7	Pvan04a	16S rDNA (long)	Litopenaeus vannamei (KT596762)	100% (519 nt/519 nt)
	P. vannamei 8	Pvan04b	16S rDNA (long)	Litopenaeus vannamei (KT596762)	100% (519 nt/519 nt)
Penaeus monodon	*P. monodon* 1	Pmon01a	16S rDNA (long)	Penaus monodon (MF563558)	100% (519 nt/519 nt)
			COI GenBank	Penaeus monodon (LC121767)/<br>P. monodon (MK430656)	100% (469/469)/<br>99.85% (657 nt/658 nt)
			COI BOLD	Penaeus monodon	99.85%
	P. monodon 2	Pmon01b	16S rDNA (long)	Penaus monodon (MF563558)	100% (519 nt/519 nt)
	P. monodon 3	Pmon02a	16S rDNA (long)	Penaus monodon (EU105473)/<br>P. monodon (AF217843)	100% (470 nt/470 nt)/<br>99.81% (519 nt/520 nt)
			COI GenBank	Penaeus mododon (MK430659)	100% (658 nt/658 nt)
			COI BOLD	Penaeus monodon	100%
	P. monodon 4	Pmon02b	16S rDNA (long)	Penaus monodon (EU105473)/<br>P. monodon (AF217843)	100% (470 nt/470 nt)/<br>99.81% (519 nt/520 nt)

continued

Species	Specimen	Original sample designation	Gene marker (and database)	First GenBank hit (sorted by Maximum Percent Identity/sorted by Maximum Score)[a] /First BOLD hit	GenBank identity of first hit (sorted by Maximum Percent Identity/sorted by Maximum Score)[a] / BOLD similarity of first hit
	P. monodon 5	Pmon03a	16S rDNA (long)	Penaus monodon (MF563558)	100% (519 nt/519 nt)
	P. monodon 6	Pmon03b	16S rDNA (long)	Penaus monodon (MF563557)	100% (519 nt/519 nt)
	P. monodon 7	Pmon04a	16S rDNA (long)	Penaus monodon (MF563558)	100% (519 nt/519 nt)
	P. monodon 8	Pmon04b	16S rDNA (long)	Penaus monodon (MF563558)	99.81% (518 nt/519 nt)
**Penaeidae sp.**	Penaeidae sp. 1	Pnotial01a	16S rDNA (short)	Heika japonica (AY452770)/<br>Penaeus duorarum (MG001106)	98.44% (63 nt/64 nt)/<br>97.43% (265 nt/272 nt)
	Penaeidae sp. 2	Pnotial01b	16S rDNA (short)	Heika japonica (AY452770)/<br>Penaeus duorarum (MG001106)	98.44% (63 nt/64 nt)/<br>97.43% (265 nt/272 nt)
			COI GenBank	Farfantepenaeus notialis (MF287098)/<br>Penaeus notialis (X84350)	97.07% (597 nt/615 nt)/<br>95.59% (628 nt/657 nt)
			COI BOLD	Farfantepenaeus notialis	97.06%
	Penaeidae sp. 3	Pnotial01c	16S rDNA (short)	Heika japonica (AY452770)/<br>Penaeus duorarum (MG001106)	98.44% (63 nt/64 nt)/<br>97.43% (265 nt/272 nt)
Penaeus* (*Fenneropenaeus*) *indicus	*P. indicus* 1	Pindic01	16S rDNA (short)	Fenneropenaeus indicus (MH094390)	100% (272 nt/272 nt)
			COI GenBank	Fenneropenaeus indicus (KC409393)	100% (637 nt/637 nt)
			COI BOLD	Fenneropenaeus indicus	100%
	P. indicus 2	Penaeid03a	16S rDNA (long)	Fenneropenaeus indicus (KX462904)	100% (519 nt /519 nt)
			COI GenBank	Fenneropenaeus indicus (KP970376)	99.66% (587 nt/589 nt)/<br>99.54 (655 nt/658 nt)
			COI BOLD	Fenneropenaeus indicus	99.83%
Penaeus* (*Fenneropenaeus*) *merguiensis	*P. merguiensis*	Penaeid03d	16S rDNA (long)	Fenneropenaeus merguiensis (KP637168)	100% (510 nt/510 nt)/<br>99.42% (516 nt/519 nt)
			COI GenBank	Penaeus indicus (AF284431) +<br>Fenneropenaeus merguiensis (KC409384)	99.39% (654 nt/658 nt) +<br>99.69% (646 nt/648 nt)
			COI BOLD	Fenneropenaeus merguiensis	99.83%
Parapenaeopsis stylifera	*P. stylifera* 1	Penaeid01a	16S rDNA (long)	Parapenaeopsis stylifera (MH045067)	99.4% (495 nt/498 nt)
			COI GenBank	Parapenaeopsis stylifera (KY316145)/<br>P. aff. sculptilis (KX399432)	100% (573 nt/573 nt)/<br>99.84% (623 nt/624 nt)
			COI BOLD	Parapenaeopsis stylifera	100%
	P. stylifera 2	Penaeid01e	16S rDNA (long)	Parapenaeopsis stylifera (MH045067)	99.8% (497 nt/498 nt)
			COI GenBank	Parapenaeopsis stylifera (KY316145)/<br>P. aff. sculptilis (KX399432)	100% (573 nt/573 nt)/<br>99.84% (623 nt/624 nt)
			COI BOLD	Parapenaeopsis stylifera	100%
Xiphopenaeus kroyeri	*X. kroyeri*	Xkroy01	16S rDNA (short)	Xiphopenaeus kroyeri (KY449069)	100% (272 nt/272 nt)
			COI GenBank	Xiphopenaeus kroyeri (MH300662)/<br>X. kroyeri (KX196599)	100% (473 nt/473 nt)/<br>99.5% (602 nt/605 nt)

			COI BOLD	Xiphopenaeus kroyery	99.81%

continued

Species	Specimen	Original sample designation	Gene marker (and database)	First GenBank hit (sorted by Maximum Percent Identity/sorted by Maximum Score)[a] /First BOLD hit	GenBank identity of first hit (sorted by Maximum Percent Identity/sorted by Maximum Score)[a] / BOLD similarity of first hit
Metapenaeus dobsoni	*M. dobsoni* 1	Mdobs01	16S rDNA (long)	Metapenaeus tenuipes (MF581793)	95.39% (497 nt/521 nt)
			COI GenBank	Metapenaeus dobsoni (KF453214)/ M. dobsoni (KJ879280)	100% (598 nt/958 nt)/ 99.39% (654 nt/658 nt)
			COI BOLD	Metapenaeus dobsoni	100%
	M. dobsoni 2	Penaeid01c	16S rDNA (long)	Metapenaeus tenuipes (MF581793)	95.39% (497 nt/521 nt)
	M. dobsoni 3	Penaeid02b	16S rDNA (long)	Metapenaeus tenuipes (MF581793)	96.78% (499 nt/521 nt)
	M. dobsoni 4	Penaeid02a	16S rDNA (long)	Metapenaeus tenuipes (MF581793)	96.78% (499/521)
			COI GenBank	Metapenaeus dobsoni (KM508833)	99.85% (657 nt/658 nt)
			COI BOLD	Metapenaeus dobsoni	99.85
***Metapenaeus* sp.**	*Metapenaeus* sp. 1	Penaeid01d	16S rDNA (long)	Metapenaeus affinis (MK511443)/ M. affinis (NC_039179)	100% (455 nt/455 nt)/ 99.81% (518 nt/519 nt)
			COI GenBank	Metapenaeus monoceros (MK986635)/ M.affinis (NC_039179)	99.82% (566 nt/567 nt)/ 98.18% (646 nt/658 nt)
			COI BOLD	Metapenaeus affinis	98.17%
	Metapenaeus sp. 2	Penaeid01b	16S rDNA (long)	Metapenaeus affinis (MK511443)/ M. affinis (NC_039179)	100% (455 nt/455 nt)/ 99.81% (518 nt/519 nt)
**Solenoceridae**					
Pleoticus muelleri	*P. muelleri* 1	Pmuel02a	16S rDNA (long)	Pleoticus muelleri (MF490231)	99.81% (514 nt/515 nt)
	P. muelleri 2	Pmuel02b	16S rDNA (short)	Pleoticus muelleri (MH300634)	100% (270 nt/270 nt)
			COI GenBank	Pleoticus muelleri (NC_039964)	100% (658 nt/658 nt)
			COI BOLD	Pleoticus muelleri	100%
	P. muelleri 3	Pmuel02c	16S rDNA (short)	Pleoticus muelleri (MH300634)	100% (270 nt/270 nt)
**Aristaeidae**					
Aristaeopsis edwardsiana	*A. edwardsiana* 1	Aedwards01a	16S rDNA (short)	Aristaeopsis edwardsiana (JX403854)	98.89% (268 nt/271 nt)
			COI GenBank	Aristeomorpha foliacea (NC_039153)	84.91% (557 nt/656 nt)
			COI BOLD	Aristaeopsis edwardsiana	99.68%
	A. edwardsiana 2	Aedwards01b	16S rDNA (short)	Aristaeopsis edwardsiana (JX403854)	98.52% (267 nt/271 nt)
			COI GenBank	Aristeomorpha foliacea (NC_039153)	85.06% (558 nt/656 nt)
			COI BOLD	Aristaeopsis edwardsiana	99.45%
	A. edwardsiana 3	Aedwards01c	16S rDNA (short)	Aristaeopsis edwardsiana (JX403854)	99.26% (269 nt/271 nt)
**Crangonidae**					
Crangon crangon	*C. crangon* 1	Ccran01a	16S rDNA (long)	Crangon crangon (KT952496)	100% (464 nt/464 nt)
			COI GenBank	Crangon crangon (KT208904)	100% (650 nt/650 nt)
			COI BOLD	Crangon crangon	100%
	C. crangon 2	Ccran01b	16S rDNA (long)	C. crangon (KT952496)	100% (464 nt/464 nt)

continued

**Species**	**Specimen**	**Original sample designation**	**Gene marker (and database)**	**First GenBank hit (sorted by Maximum Percent Identity/sorted by Maximum Score)[a] /First BOLD hit**	**GenBank identity of first hit (sorted by Maximum Percent Identity/sorted by Maximum Score)[a] / BOLD similarity of first hit**
**Pandalidae**					
Heterocarpus sp.	*Heterocarpus* sp. 1	Hreed01a	16S rDNA (long)	Heterocarpus sp. (MH300626)/ Heterocarpus sp. (MH300640)	100% (243 nt/243 nt)/ 99.79% (479 nt/480 nt)
			COI GenBank	Heterocarpus sp. (MH300659)	100% (477 nt/477 nt)
			COI BOLD	Heterocarpus sp.	100%
	Heterocarpus sp. 2	Hreed01b	16S rDNA (long)	Heterocarpus sp. (MH300640)	99.79% (479 nt/480 nt)
***Pandalus* sp.**	*Pandalus* sp. 1	Pbor01a	16S rDNA (short)	Pandalus borealis (MH300624) + Pandalus eous (LC121745)	100% (227 nt/227 nt)
	Pandalus sp. 2	Pbor01b	16S rDNA (short)	Pandalus borealis (MH300624) + Pandalus eous (LC121745)	100% (265 nt/265 nt)
			COI GenBank	Pandalus borealis (KY018757)/ Pandalus borealis (KY018893)	99.70% (656 nt/658 nt)/ 100% (227 nt/227 nt)
			COI BOLD	Pandalus borealis + Pandalus eous	99.85% + 99.84%
	Pandalus sp. 3	Pbor01c	16S rDNA (short)	Pandalus borealis (MH300624) + Pandalus eous (LC121745)	100% (265 nt/265 nt)
**Palaemonidae**					
Macrobrachium rosenbergii/dacqueti	*M. rosenbergii/dacqueti*	Mrosenb01	16S rDNA (short)	Macrobrachium rosenbergii (MF402019)	100% (255 nt/255 nt)
			COI GenBank	Macrobrachium rosenbergii (AY554326)/ M. rosenbergii (MF563572)	100% (593 nt/593 nt)/ 99.85% (657 nt/658 nt)
			COI BOLD	Macrobrachium rosenbergii	100%
**Cambaridae**					
Procambarus clarkii	*P. clarkii* 1	Pclar01a	16S rDNA (short)	Pcrocambarus clarkii (MH300651)	99.61% (257 nt/258 nt)
			COI GenBank	Procambarus clarkii (MF170536)	100% (582 nt/582 nt)
			COI BOLD	Procambarus clarkii	100%
	P. clarkii 2	Pclar01c	16S rDNA (short)	Procambarus clarkii (KJ645830)	100% (265 nt/265 nt)

[a]) If query coverage was < 100%, the first hit sorted by Maximum Score is also displayed in addition of the first hit sorted by Maximum Percent Identity.
